# Physical strength, body composition, and G-test results of air force cadets based on nutrition knowledge differences

**DOI:** 10.1038/s41598-024-53600-4

**Published:** 2024-02-07

**Authors:** Jun-Young Sung, Kyu-Lim Lee

**Affiliations:** 1 Institute of Human Convergence Health Science, Gachon University, Hambakmoe-ro, Yeonsu-gu, Incheon, South Korea; 2 Division in Anatomy and Developmental Biology, Department of Oral Biology, Human Identification Research Institute, BK21 FOUR Project, Yonsei University College of Dentistry, 50–1 Yonsei-ro, Seodaemun-gu, Seoul, 03722 South Korea

**Keywords:** Health care, Health occupations

## Abstract

The Gravitational Acceleration Test (G-test) is a demanding, and sophisticated high-intensity physical activity, greatly influenced by individual body composition and physical strength. This study analyzed the general nutrition knowledge questionnaire (GNKQ) responses of Air Force cadets to identify the relationship between nutrition knowledge, body composition, physical activity, physical strength, and gravity-induced loss of consciousness. Based on the G-test results, 105 fourth-year Air Force cadets were divided into two groups: GP (G-test pass group) and GF (G-test fail group). The analysis items were GNKQ responses, body composition, and physical strength analysis, based on which independent sample t-tests , and logistic regression analysis were conducted. Physical activity according to the G-test results was statistically higher in the GP compared to the GF (vigorous activity reps/week, *p* = 0.017; mins/day, *p* = 0.011). The GP Group showed a statistically high GNKQ score compared to the GF Group: Overall (*p* = 0.003), Section 1 (*p* < 0.001), and Section 2 (*p* = 0.002). Based on this study, it can be deduced that analyzing the effect G-test through continuous research over the next years and applying them to physical training will have a greater impact on the cadets’ increased physical strength and their success on the G-test.

## Introduction

Sports nutrition involves the fields of sports medicine, sports science, dietetics, cultural influences, and even popular media^1^. It is not merely a concept concerning diet; it encompasses an interdisciplinary approach to the various aspects of sports that affect athletes, such as nutrition, exercise, rest, and recovery between competition, preparation, and finish^2^. Recently, the research field and target of sports nutrition have been widely expanded, and the military is known to be a very suitable target for the application of sports nutrition.

Among the success factors of military operations, nutritional management of combat soldiers is a key factor^3^, and adequate nutrition is crucial for maintaining physical and mental functions, preventing disease, and performing tasks^4^. Specifically, Air Force fighter pilots need to maintain their mental and physical functions as they are vulnerable to extreme physiological environments^5^ . In aerial environments, pilots are exposed to various risk factors, including gravity-induced loss of consciousness (G-LOC), hypoxia, cognitive dissonance, hearing loss, and flight illusion. The G-test is a high-intensity physical activity that is greatly influenced by individual body composition and physical strength^6^, Thus, strong physical strength and sufficient nutritional intake are essential^7^.

To provide nutrition suitable for energy consumption, a survey on participants’ energy intake and eating habits should be conducted, which should include survey items like individual eating habits factors such as culture, beliefs, self-efficacy, and nutritional knowledge among others^8^. Among them, nutritional knowledge is expressed by scoring individual nutritional knowledge levels, which can have a direct effect on eating habits. Thus, the general nutrition knowledge questionnaire (GNKQ), a nutritional knowledge measurement tool, is being used internationally across various fields.

**Figure 1:**
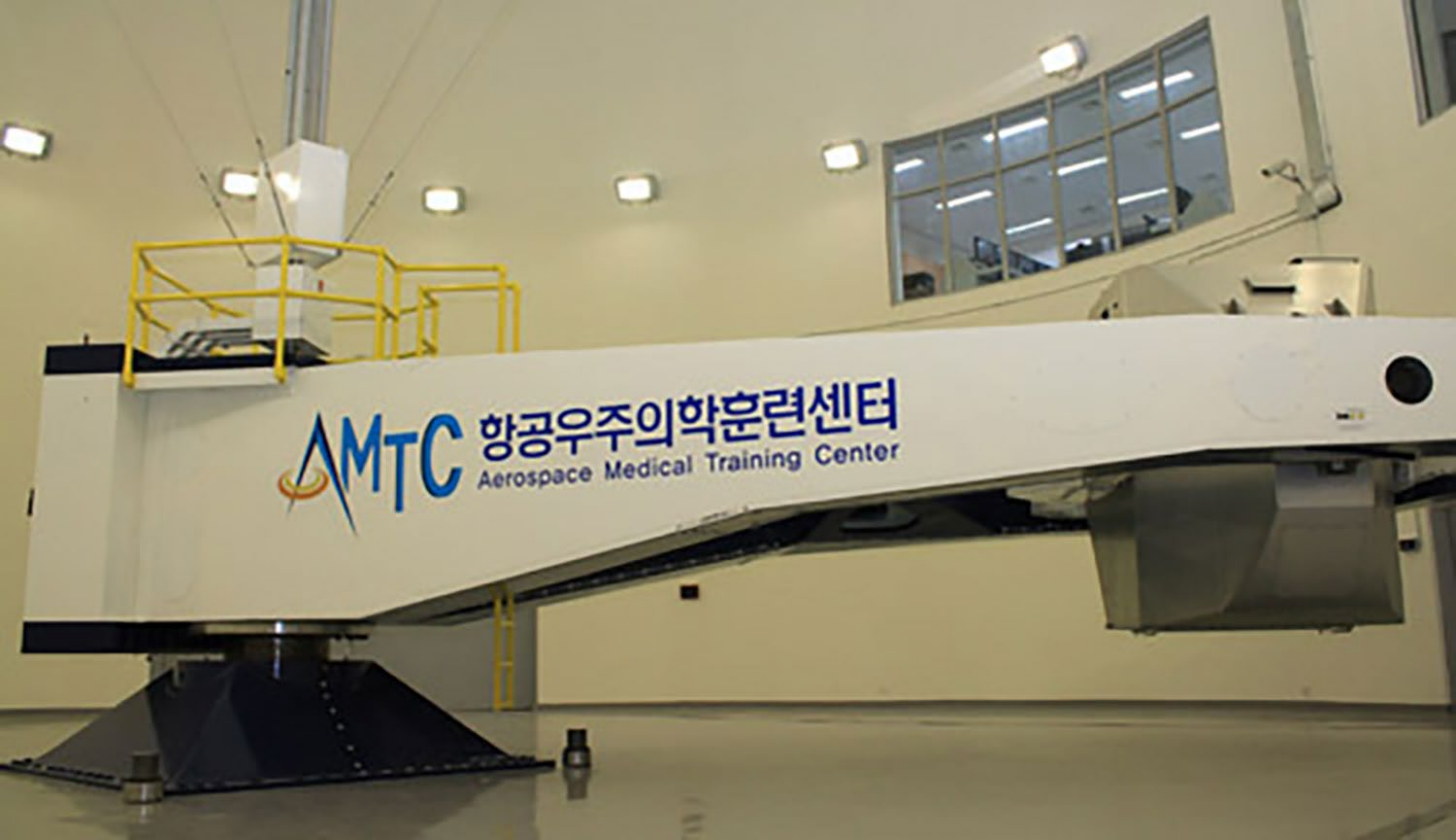
The high-speed centrifugal motion gondola (ETC, USA) used in this study.

The GNKQ was first developed and verified for the adult population of the United Kingdom in the 1990s^9^, and was revised to GNKQ-R in 2016. Nutritional knowledge is only one of many factors influencing food choices, the fact remains unchanged that nutritional knowledge is pivotal in choosing a healthy diet^10^ . In many previous studies, the GNKQ test was conducted on soldiers^11–13^. However, despite the need for research on the combat capability, to improve the G-resistance and health maintenance of air force pilots, prior research on pilots is insufficient (Fig. 1).

Therefore, this study identified and applied related variables to improve the G-resistance of air force pilots and analyze the GNKQ of air force cadets to identify the relationship between nutrition knowledge, body composition, physical activity, physical strength, and G-LOC. Through this study, we intend to improve physical activity among the cadets through scientific sports activities and establish a database for the nutrition education of cadets and pilots.

## Results

### G-test passes and fails according to physical strength characteristics

The difference in body composition according to the G-test result is that the GP group weights (GP, 72.24 ± 7.37; GF, 68.79 ± 8.92; *p* = 0.040) and BMI (GP, 24.05 ± 1.48; GF, 23.08 ± 1.51; *p* = 0.003) showed a statistically high difference compared to the GF group. Interestingly, skeletal muscle mass was higher for the GP than the GF group but did not show a statistically significant difference (Table 1).

**Table 1:** Participant characteristics.

Variables	GP (n = 72)		GF (n = 43)		F	t	*p*
	Mean	SD	Mean	SD			
Height (cm)	173.13	4.91	172.28	8.57	25.456	0.641	0.523
Body weight (kg)	72.24	7.37	68.79	8.92	4.935	2.082	0.040*
Skeletal muscle mass (kg)	33.90	5.37	31.71	5.51	1.490	1.917	0.058
Skeletal muscle mass (%)	46.68	3.55	45.88	3.02	0.006	1.131	0.261
Body fat mass (kg)	12.57	2.84	12.52	2.35	0.748	0.083	0.934
Body fat mass (%)	17.69	5.10	18.49	4.28	0.001	− 0.783	0.435
Body mass index (kg/m^2^)	24.05	1.48	23.08	1.51	0.065	3.088	0.003*

Values expressed *p < 0.05 vs. GP, G-test pass group; GF, G-test fail group.

**Figure 2:**
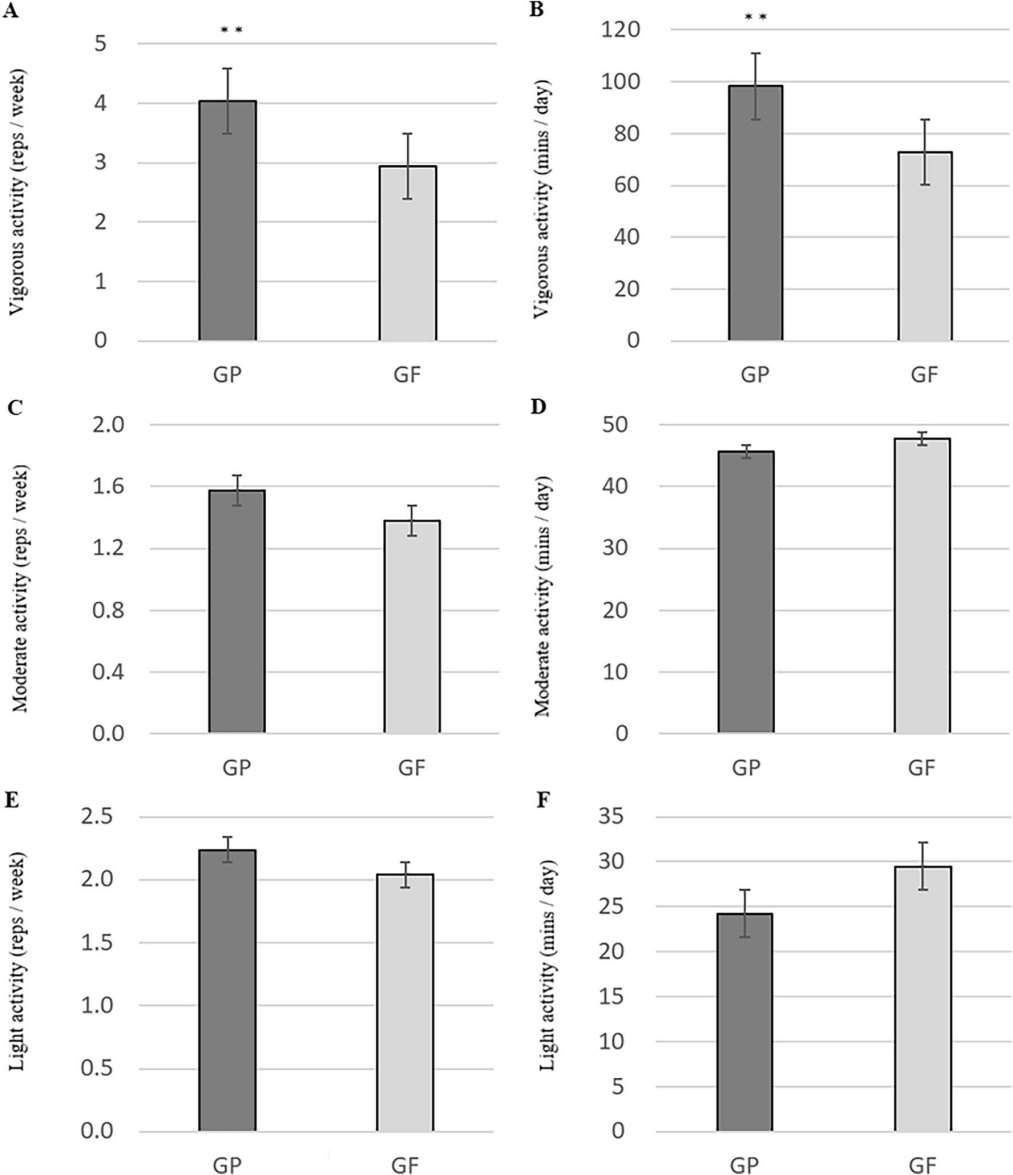
Change in physical activity by G-test result: (**A**) Vigorous activity (reps/week), (**B**) Vigorous activity (mins/day), (**C**) Moderate activity (reps/week), (**D**) Moderate activity (mins/day), (**E**) Light activity (reps/week), (**F**) Light activity (mins/day); GP, G-test pass group; GF, G-test fail group.

The difference in physical activity according to the G-test results was statistically higher for the GP group regarding vigorous activity: reps/week (GP, 4.07 ± 1.44; GF, 2.95 ± 1.01; *p* = 0.017) and vigorous activity: mins/day (GP, 97.54 ± 54.32; GF, 75.32 ± 56.25; *p* = 0.011) (Fig. 2)*.*

The physical strength evaluation results according to the G-test results did not show a statistically significant difference in all factors (Table 2).

**Table 2:** Physical strength test results by G-test result.

Variables	GP (n = 72)		GF (n = 43)		F	t	*p*
	Mean	SD	Mean	SD			
3 km running (min sec)	11.44	2.35	10.32	4.29	15.278	1.719	0.089
Sit-up (reps)	93.80	20.24	90.45	21.05	0.061	0.840	0.403
Push-up (reps)	94.25	22.80	90.59	25.58	0.140	0.786	0.433

Values expressed; GP, G-test pass group; GF, G-test fail group.

### G-test passes and fails according to nutrition knowledge differences

The difference in GNKQ score according to the G-test result was the following: the GP Group showed a statistically high score compared to the GF Group: Overall (GP, 66.68 ± 5.50; GF, 60.12 ± 6.35; *p* = 0.003), Sect. 1 (GP, 11.24 ± 2.44; GF, 8.97 ± 1.65; *p* < 0.001), and Section 2 (GP, 25.78 ± 2.81; GF, 23.94 ± 2.51; *p* = 0.002). Sections 3 and 4 also showed higher scores for the GP group than the GF group, without any statistically significant difference (Table 3).

**Table 3:** GNKQ score by G-test result.

Variables	Nutrition knowledge section (max possible score)														
	Overall (88)			Section 1 (18)			Section 2 (36)			Section 3 (13)			Section 4 (21)		
	Mean	SD	%	Mean	SD	%	Mean	SD	%	Mean	SD	%	Mean	SD	%
GP (n = 72)	65.68	5.50	74.6	11.24	2.44	62.4	25.78	2.81	71.6	11.03	2.42	84.8	17.61	1.87	83.8
GF (n = 43)	60.12	6.35	68.3	8.97	1.65	49.8	23.94	2.51	66.5	10.09	2.83	83.8	17.09	1.93	81.4
F	0.065			8.302			0.001			1.012			0.012		
t	3.088			4.855			3.210			1.744			1.309		
*p*	0.003**			< 0.001***			0.002**			0.084			0.193		
*Effect size*^*a*^	0.9			1.1			0.7			0.4			0.3		

Values expressed **p < 0.01, ***p < 0.001vs GP by t-test; GP, G-test pass group; GF, G-test fail group; Section  1: dietary recommendation; Section 2: food groups; Section 3: healthy food choices; Section 4: diet, disease, and weight associations;^***a***^Cohen's effect size.

GNKQ: general nutrition knowledge questionnaire.

The logistic regression analysis result of the GNKQ score according to the G-test result is Section 1 (OR, 0.174; *p* = 0.026); Sect. 2 (OR, 0.205; *p* = 0.047) showed statistical significance levels in the GP and the GF groups (Table 4).

**Table 4:** Logistic Regression Analysis of the GNKQ Score and G-test results.

Variables		B	S.E	OR	95%CI	*P*
G-test result (Pass or Fail)	Overall	1.291	0.768	3.636	(0.807 ~ 16.383)	0.093
	Section 1	− 1.746	0.782	0.174	(0.038 ~ 0.808)	0.026*
	Section 2	− 1.583	0.797	0.205	(0.043 ~ 0.979)	0.047*
	Section 3	− 1.331	0.776	0.264	(0.058 ~ 1.209)	0.086
	Section 4	− 1.386	0.801	0.250	(0.052 ~ 1.201)	0.083
	* − 2LL* = *200.470 , NagelKeeke R2* = *0.354, Hosmer & Lemeshow test: χ2* = *14.854(p* = *0.062)*					

A significant difference * *p* < .05; Section 1: dietary recommendation; Section 2: food groups; Section 3: healthy food choices; Section 4: diet, disease and weight associations.

GNKQ: general nutrition knowledge questionnaire.

## Discussion

This study analyzed the GNKQ of air force cadets, and investigated the relationship between nutritional knowledge, body composition, physical activity, physical strength, and G-LOC.

The G-test is a high-intensity physical activity, greatly influenced by individual body composition and physical strength. The G-test results for each body composition showed no significant difference in all factors other than weight and BMI. However, the GP group had higher skeletal muscle mass and lower body fat mass and body fat ratio than the GF group. Koo’s (2002) study highlighting that skeletal muscle mass is more necessary to pass the G-test supports the findings^14^. It has been reported that increased muscle mass increases peripheral resistance and increases blood pressure, preventing the loss of consciousness^15^. Furthermore, muscles strongly contract blood like anti-G suits, increasing blood discharge from the left ventricle of the heart and reducing blood flow to the lower extremities, thereby improving G-resistance by facilitating blood supply to the brain. Increased muscle contraction and respiratory muscle development enhance the effectiveness of anti-G training maneuvers to prevent loss of consciousness^7,16^. Metzler (2020) in their study reported the importance to design a program that considers the balance between muscle training and aerobic training^17^. Therefore, it is important to design the training programs of the Air Force Academy based on findings of previous studies.

According to prior studies, vigorous physical activity is essential for pilots to maintain an appropriate body composition^2^ . In this study, the GP group showed statistically higher results in active physical activity (reps and time) than the GF group. Thus, the cadets who passed the G-test were participating in more physical activities and striving to improve their physical strength.

Air Force cadets need special attention to their diet and physical activity to maintain proper body composition required for pilots. According to our finding, the GP group scored higher in the nutritional knowledge questionnaire than the GF group. However, as only nutritional knowledge questionnaires and physical strength evaluation results are used, calculating the actual intake, activity, and energy metabolic rate of cadets using measuring equipment is necessary for future studies.

Nutrition knowledge has various effects on an individual’s dietary intake^18^. Considering the general knowledge behavior model, we found that individuals are more likely to make informed decisions about their health if they understand the benefits of nutrition^10^. Previous studies involving athletes and soldiers engaged in various sports, have studied the relationship between nutritional knowledge and dietary behavior^3,18–20^. Cadets had particularly low knowledge of daily recommendation (Sect. 1) and food group (Sect. 2). Consequently, there is a lack of professional nutrition education, and it is necessary to introduce a nutrition education program for cadets.

Nutrition education programs focus on providing evidence-based information on energy and large-scale nutrient requirements and should include individualized approaches. Nutrition education, which is directly linked to dietary intake, can act as a motivation for behavioral changes and limit the impact of other external factors on intake, such as body composition evaluation^21^. Additionally, to provide nutrition education for military cadets and pilots with special status as soldiers, an understanding of their activity characteristics and eating habits should be preceded.

Physical function and instantaneous situational judgment are important factors that cannot be eliminated on the battlefield where extreme situations are exchanged. Food is energy to humans and is essential for survival. This study investigated the relationship between G-LOC and the nutritional knowledge of each prospective pilot, which has not been attempted before. The findings do not indicate that cadets with only low nutritional knowledge were susceptible to G-LOC. Having a fair amount of knowledge about food is related to one’s eating habits; it is considered that the continuous practice of eating habits and daily life (sports activities, etc.) affected the G-test results.

The aerospace industry will continue to develop with the development of science and technology. Therefore, research on aircrafts and the physical and mental states of pilots should be regularly conducted. The Air Force Academy will need systematic nutrition education and management to maintain body composition of cadets to increase the acceptance rate of G-test for cadets. Additionally, based on these results, further research is needed to consider preserving the life of pilots and improving their quality of life by investigating eating habits, nutritional knowledge, nutrition intake, activities, and energy metabolism.

## Methods

### Participants

For this study, participants were recruited in March 2022. A hundred and five male cadets among the seniors of the Republic of Korea Air Force Academy, who conducted the 2022 G-test, participated in this study; however, those who did not wish to participate, were injured, or did not conduct G-test were excluded from the study. All participants performed the same schedule (department work, meals, sleep, training, etc.). The whole process was conducted for three months between April 1 and July 30, 2022, The number of samples in this study was analyzed using the G*power3.0 program^22^.

Before the study began, the intention to participate was confirmed face-to-face for cadets conducting G-TEST. All participants were given a detailed description of the study’s purpose, methods, and risks involved and were informed that they could withdraw from the experiment at any time, without any repercussions. Subsequently, they signed an informed consent form. All study procedures were approved by the Ethics Committee of the Air Force Aerospace Medical Center Institution Bioethics Committee (ASMC-22-IRB-003). This study procedure followed the principles of the Declaration of Helsinki.

The participants were classified into GP (30 s G-test pass group) and GF (30 s G-test fail group) according to the G-test results. Table 1 shows the physical characteristics of the study subjects.

### Institutional review board statement

The study was conducted according to the guidelines of the Declaration of Helsinki and approved by the Air Force Aerospace Medical Center Institution Bioethics Committee (ASMC-22-IRB-003; date of approval, 16/06/22). Informed Consent Statement: Informed consent was obtained from all the study participants.

## Measurement instrument

### G-test

The G-test was conducted for two months between May 1 and June 30, 2022. The G-test is measured using a high-speed centrifugal motion gondola (ETC, USA) located at the Air Force Aerospace Medical Center (Fig. 1). The participants are seated in a cockpit-type seat and a G-test is performed for 30 s at 5 G acceleration. Shortly after starting, the gondola starts spinning at a speed of 0.8 G, accelerating to 5 G as soon as the participants pull the lever directly. The end measurement stops 30 s after the start or when the participant pushes the lever or loses consciousness due to G-LOC.

### Physical strength characteristics measurement

Physical strength characteristics measurement was conducted for three months between April 24 and June 24, 2022. Body composition measurement was performed five days before the G-test measurement. The participants refrained from high-intensity activities and had sufficient sleep the previous day; measurement of body composition was conducted at 08 a.m. on an empty stomach. Weight (kg), skeletal muscle mass (kg), body fat mass (kg), body fat percentage (kg), and body mass index (kg/m2) were measured using InBody 720 (Biospace Co., Ltd., Seoul, Republic of Korea); additionally, height (cm) was measured using a body system.

In the afternoon, a physical strength test was conducted according to the Ministry of National Defense’s physical fitness test event. The tests included running three kilometers, and performing push-ups, and sit-ups.

Questions about physical activity (International Physical Activity Questionnaire – abbreviated for this study) were also used. The survey questions were based on a short questionnaire about international physical activity^23^. The reliability of the questionnaire was evaluated through pre-experimental execution performed by the researchers. Pre-experimental data indicated that the test reliability coefficient of the survey is suitable (r = 0.84–0.96).

### General nutrition knowledge questionnaire

The survey was conducted for three months between April and June, 2022. Despite an existing sport general knowledge questionnaire (SGNKQ) for athletes, a general knowledge questionnaire (GNKQ) was used because the participants were cadets in the air force academy.

The GNKQ used was the translated GNKQ-R revised by Kliemann, Wardle, Johnson, and Croker^24^ into Korean. The GNKQ-R comprised 88 items and four sections by reviewing the contents of the UK and international nutrition guidelines. One point was given for each correct answer and zero points were given for “uncertain” or incorrect responses. The overall score (out of 88) was marked as the correct answer rate.

### Statistical analysis

Data organization and statistical analysis were conducted for a month from July 1 to July 30, 2022. For data processing, the mean and standard deviation of all data were calculated using the SPSS version 22 (IBM Corp, Armonk, NY, USA) statistical program. An independent sample *t*-test was used to analyze the difference in GNKQ, physical activity amount, and physical strength based on G-test results. Additionally, logistic regression analysis was conducted to confirm the relationship between GNKQ and the success or failure of G-test training. The significance level of all hypothesis verification is *p* < 0.05.

## Data Availability

The data presented are available on request from the corresponding author.

## References

[1] Vitale K, Getzin A (2019). Nutrition and supplement update for the endurance athlete: Review and recommendations. Nutrients.

[2] Lee BK, Lee BK (2021). Prevalence of ischemia, health-related quality of life, medical use and expenses by physical activity and ischemia status in Korean adults. Exerc. Sci..

[3] Kullen CJ, Farrugia JL, Prvan T, O’Connor HT (2016). Relationship between general nutrition knowledge and diet quality in Australian military personnel. Br. J. Nutr..

[4] Montain SJ, Carvey CE, Stephens MB (2010). Nutritional fitness. Mil. Med..

[5] Shin SH (2018). A correlation pilot-study of F-15/16 pilots’ ACTN-3, G-tolerance, and body compositions. Exerc. Sci..

[6] Jeong DH, Lee DR, Lee KL, Sung JY (2023). Gravitational acceleration test results according to functional movement screen and morphological symmetry results of air force cadets. Symmetry.

[7] Kim I, Jeong D-H, Sung JY, Kim K-S (2022). Analysis of G-test results according to fatigue, physical fitness and body composition of Air Force cadets using smart watches. Exerc. Sci..

[8] Birkenhead KL, Slater G (2015). A review of factors influencing athletes’ food choices. Sports Med..

[9] Parmenter K, Wardle J (1999). Development of a general nutrition knowledge questionnaire for adults. Eur. J. Clin. Nutr..

[10] Worsley A (2002). Nutrition knowledge and food consumption: Can nutrition knowledge change food behaviour?. Asia Pac. J. Clin. Nutr..

[11] Bovill ME, Tharion WJ, Lieberman HR (2003). Nutrition knowledge and supplement use among elite US Army soldiers. Mil. Med..

[12] Herzman-Harari S, Constantini N, Mann G, Lencovsky Z, Stark AH (2013). Nutrition knowledge, attitudes, and behaviors of Israeli female combat recruits participating in a nutrition education program. Mil. Med..

[13] Kullen CJ, Iredale L, Prvan T, O’Connor HT (2016). Evaluation of general nutrition knowledge in Australian military personnel. J. Acad. Nutr. Diet..

[14] Koo MS (2002). Physical fitness program to enhance aircrew G tolerance. J. Korea. Exerc. Sci. Acad..

[15] Green ND, Ford SA (2006). G-induced loss of consciousness: Retrospective survey results from 2259 military aircrew. Aviat. Space. Environ. Med..

[16] Jagim AR (2021). The influence of sport nutrition knowledge on body composition and perceptions of dietary requirements in collegiate athletes. Nutrients.

[17] Metzler MM (2020). G-LOC due to the push-pull effect in a fatal F-16 mishap. Aerosp. Med. Hum. Perform..

[18] Heaney S, O’Connor H, Michael S, Gifford J, Naughton G (2011). Nutrition knowledge in athletes: A systematic review. Int. J. Sport. Nutr. Exerc. Metab..

[19] Devlin BL, Leveritt MD, Kingsley M, Belski R (2017). Dietary intake, body composition, and nutrition knowledge of Australian football and soccer players: Implications for sports nutrition professionals in practice. Int. J. Sport. Nutr. Exerc. Metab..

[20] Spronk I, Heaney SE, Prvan T, O’Connor HT (2015). Relationship between general nutrition knowledge and dietary quality in elite athletes. Int. J. Sport. Nutr. Exerc. Metab..

[21] Jenner SL (2018). Dietary intake of professional Australian football athletes surrounding body composition assessment. J. Int. Soc. Sports Nutr..

[22] Faul F, Erdfelder E, Buchner A, Lang A-G (2009). Statistical power analyses using G*Power 3.1: Tests for correlation and regression analyses. Behav. Res. Methods.

[23] Ammar, A., *et al*. On Behalf of The Eclb-Covid Consortium. COVID-19 home confinement negatively impacts social participation and life satisfaction: A worldwide multicenter study. *Int. J. Environ. Res. Public Health*, **17**(17), 6237 (2020). 10.3390/ijerph17176237. 10.3390/ijerph17176237 PMC7503681 32867287

[24] Kliemann N, Wardle J, Johnson F, Croker H (2016). Reliability and validity of a revised version of the general nutrition knowledge questionnaire. Eur. J. Clin. Nutr..

